# *In-vitro *Studies on *Calotropis procera *Leaf Extracts as Inhibitors of Key Enzymes Linked to Diabetes Mellitus

**Published:** 2016

**Authors:** Mutiu Idowu Kazeem, Ayuva Mercy Mayaki, Bimpe Folashade Ogungbe, Anthony Babajide Ojekale

**Affiliations:** *Department of Biochemistry, Faculty of Science, Lagos State University, PMB 0001, Ojo, Lagos, Nigeria.*

**Keywords:** *Calotropis procera*, Diabetes mellitus, A-amylase, A-glucosidase, Tannins

## Abstract

The side effects associated with the usage of synthetic antidiabetic drugs make it imperative to search for alternative drugs from medicinal plants. Therefore, this study was aimed at evaluating the α-amylase and α-glucosidase inhibitory potential of *Calotropis procera* leaf, as well as its possible mode of inhibiting these enzymes. Acetone, aqueous and ethanolic extracts of *C. procera* leaf was subjected to standard enzymes’ inhibitory assay *in-vitro* using porcine pancreatic α-amylase and rat intestinal α-glucosidase. Results obtained showed that out of all the extracts tested, ethanolic and aqueous extracts possessed the best inhibition of α-amylase (IC_50_ 7.80 mg/mL) and α-glucosidase (3.25 mg/mL) respectively. The kinetic analysis of the mode of inhibition of these enzymes by the leaf extracts of *C. procera*, revealed that these extracts inhibited both enzymes in a non-competitive manner. It is speculated that the α-amylase and α-glucosidase inhibitory properties of leaf extracts of *C. procera* may be due to the presence of some phytochemicals such as flavonoids, tannins and saponins in the plant. It can be concluded from this study that the *Calotropis procera* extracts could serve as source of antidiabetic agents which may act through the inhibition of carbohydrate hydrolyzing enzymes, α-amylase and α-glucosidase.

## Introduction

The search for the discovery of antidiabetic drugs from medicinal plants is an important strategy that is required to combat the widespread nature of diabetes mellitus among all populations of the world (1). This is due to the fact that present synthetic drugs are bedevilled with many limitations ranging from limited efficacy and several side effects such as hypoglycaemia, weight gain and chronic tissue damage (2, 3). Consequent upon this, several plants with folkloric usage as antidiabetics are being verified with the aid of scientific methods. Some of these plants include *Blighia sapida*,* Morus alba*,* Spondias monbin*, *Treculia africana *and* Calotropis procera* (4). 

*Calotropis procera *(giant milkweed) is a perennial, greyish-green, woody shrub with broad fleshy leaves that grows wild in the tropics and in warm temperate regions (5). The plant is found in most parts of Nigeria but most abundant in the northern part of the country (6). It is primarily harvested because of its distinctive medicinal properties (7). It is used in the treatment of pain as well as inflammation (8) and to aid digestion. The latex of the plant is used as an antidysenteric, antirheumatic, a diaphoretic, an expectorant and for the treatment of bronchial asthma and skin conditions (9). In African and Asian countries, the latex of *C. procera *is utilized as an arrow poison, molluscide, a fungicide, an anti-syphilitic, an anti-inflammatory, a purgative, as well as in the treatment of leprosy and bronchial asthma (10). The dried latex and root are used as an antidote for snake poisoning. It is also used as an abortifacient (11), and for the treatment of piles (12) and intestinal worms (13).

The previous pharmacological studies include reports of anticancer, antifungal (14) and insecticidal activities of *C. procera*. The flowers and latex of the plant exhibited hepatoprotective (15, 16), anti-inflammatory, antipyretic, analgesic, antimicrobial and larvicidal activity (17, 18). The latex of the plant is also reported to possess analgesic and wound healing activity (19, 20), as well as anti-inflammatory (21) and antimicrobial activities (22) (22), while the roots displayed anti-fertility (23) and anti-ulcer effects (24).

Though some studies have been conducted on the antidiabetic potentials of *C. procera* (25, 26) but there is dearth of information on the mechanism of antihyperglycemic action of this plant. Therefore, the aim of this study was to evaluate the α-amylase and α-glucosidase inhibitory potential of *C. procera* as a possible mechanism of hypoglycemic effect of this plant.

## Experimental

*Plant material*

The plant *Calotropis procera* leaf was collected in February 2012 at Agboroko area of Ojo, Lagos state, Nigeria. It was identified and authenticated at the Department of Botany, University of Lagos, Nigeria and voucher specimen (LUH 4719) was deposited in the University herbarium.

*Chemicals*

Porcine pancreatic α-amylase, rat intestinal α-glucosidase and paranitrophenyl-glucopyranoside (PNPG) were products of Sigma-Adrich Co., St Louis, USA while starch soluble (extra pure) was obtained from J. T. Baker Inc., Phillipsburg, USA. Other chemicals and reagents were of analytical grade and water used was glass-distilled.

*Preparation of plant extracts*

Fresh leaves of *Calotropis procera* were cut and washed with water to remove all contaminants; they were dried under room temperature and grounded to powder. The powdered leaves were divided into three portions and each portion was dissolved in either acetone, ethanol or water. They were all left to steep in covered containers for 24 h; the resulting infusions were decanted and filtered. Acetone and ethanolic extracts were evaporated in a rotary evaporator (Cole Parmer SB 1100, Shangai, China), while aqueous extract was freeze-dried using Virtis Bench Top (SP Scientific Series, USA) freeze dryer. Dried extracts were weighed and dissolved in 10% dimethylsulphoxide (DMSO) to yield a stock solution from which lower concentrations were prepared.

*Phytochemical screening*

Phytochemical compositions of the leaves were determined using the methods variously described by Trease and Evans (27) and Sofowora (6).

*α-Amylase inhibitory assay*

This assay was carried using a modified procedure of McCue and Shetty (28) and the concentration of α-amylase solution used was 0.5 mg/mL. A total of 250 µL of extract was placed in a tube and 250 µL of 0.02 M sodium phosphate buffer (pH 6.9) containing α-amylase solution was added. This solution was pre-incubated at 25 ^o^C for 10 min, after which 250 µL of 1% starch solution in 0.02 M sodium phosphate buffer (pH 6.9) was added at timed intervals and then further incubated at 25 °C for 10 min. The reaction was terminated after incubation by adding 500 µL of dinitrosalicylic acid (DNS) reagent. The tubes were then incubated in boiling water for 5 min and cooled to room temperature. The reaction mixture was diluted with 5 mL distilled water and the absorbance was measured at 540 nm using spectrophotometer. A control was prepared using the same procedure replacing the extract with 10% DMSO. The α-amylase inhibitory activity was calculated as percentage inhibition.

% Inhibition = [(Abs_control_-Abs_extracts_)/Abs_control_] x 100

Concentrations of extracts resulting in 50% inhibition of enzyme activity (IC_50_) were determined graphically.

*Mode of α-amylase inhibition*

The mode of inhibition of the enzyme by the leaf extract was conducted using the extract with the lowest IC_50_ according to the modified method described by Ali *et al*. (29). Briefly, 250 μL of the (5 mg/mL) extract was pre-incubated with 250 μL of α-amylase (0.5 mg/mL) solution for 10 min at 25 ºC in one set of tubes. In another set of tubes, α-amylase was pre-incubated with 250 μL of phosphate buffer (pH 6.9). 250 µL of starch solution at increasing concentrations (0.30–5.0 mg/mL) was added to both sets of reaction mixtures to start the reaction. The mixture was then incubated for 10 min at 25 °C, and then boiled for 5 min after addition of 500 µL of DNS to stop the reaction. The amount of reducing sugars released was determined spectrophotometrically and subsequently extrapolated using maltose standard curve to obtain reaction velocities. A double reciprocal plot (1/v versus 1/[S]) where v is reaction velocity and [S] is substrate concentration was plotted*. *The type (mode) of inhibition of alpha amylase activity by the extract was determined by analysis of the double reciprocal (Lineweaver-Burk) plot using Michaelis-Menten kinetics.

*α-Glucosidase inhibitory assay*

This assay was performed according to the method described by Kim *et al*. (30) and the concentration of α-glucosidase solution used was 1.0 U/mL. The substrate solution p-nitropheynyl glucopyranoside (pNPG) was prepared in 20 mM phosphate buffer, pH 6.9. 100 µL of α-glucosidase (E.C. 3.2.1.20) was pre-incubated with 50 µL of the different concentrations of the extracts (acetone, ethanol and water) for 10 min. Then 50 µL of 3 mM p-nitropheynyl glucopyranoside as a substrate dissolved in 20 mM phosphate buffer, pH 6.9 was then added to start the reaction. The reaction mixture was incubated at 37 ^o^C for 20 min and stopped by adding 2 mL of 0.1 M Na_2_CO_3_. The α-glucosidase activity was determined by measuring the yellow colored p-nitrophenol released from the reaction at 405 nm. A control was prepared using the same procedure replacing the extract with 10% DMSO. The results were expressed as percentage of the blank control.

Percentage inhibition calculated as 

% inhibition = [(Abs_control_-Abs_extract_)/Abs_control_] x 100

Concentrations of extracts resulting in 50% inhibition of enzyme activity (IC_50_) were determined graphically.

*Mode of α-glucosidase inhibition*

The mode of inhibition of the enzyme by the leaf extract was conducted using the extract with the lowest IC_50_ according to the modified method described by Ali *et al*. (29). Briefly, 50 μL of the (5 mg/mL) extract was pre-incubated with 100 μL of α-glucosidase solution (1.0 U/mL) for 10 min at 25 ºC in one set of tubes. In another set of tubes α-glucosidase was pre-incubated with 50 μL of phosphate buffer (pH 6.9). 50 µL of p-nitropheynyl glucopyranoside at increasing concentrations (0.63 – 2.0 mg/mL) was added to both sets of reaction mixtures to start the reaction. The mixture was then incubated for 10 min at 25 °C, and 500 µL of Na_2_CO_3_ was added to stop the reaction. The amount of reducing sugars released was determined spectrophotometrically using a paranitrophenol standard curve and converted to reaction velocities. A double reciprocal plot (1/v versus 1/[S]) where v is reaction velocity and [S] is substrate concentration was plotted*. *The mode of inhibition of α-glucosidase activity by the extract was determined by analysis of the double reciprocal (Lineweaver-Burk) plot using Michaelis-Menten kinetics.

*Statistical Analysis*

Statistical analysis was performed using GraphPad Prism 5 statistical package (GraphPad Software, USA). The data were analyzed by one way analysis of variance (ANOVA) followed by Bonferroni test. All the results were expressed as mean ± SE for triplicate determinations.

## Results

Table 1 shows the result of the phytochemical screening conducted on the various extracts of *Calotropis procera* leaves. It shows that flavonoids, reducing sugars and steroids are present in both the ethanolic and aqueous extracts while tannins was detected in acetone and aqueous extracts. However, saponin was present in the aqueous extract only. 

**Table 1:** The phytochemical composition of different extracts of *Calotropis procera* leaf

**Phytochemicals**	**Extracts inference**		
	**Acetone**	**Ethanol**	**Water**
Anthraquinones	-	-	-
Flavonoids	-	+	+
Reducing sugar	-	+	+
Saponins	-	-	+
Steroids	-	+	+
Tannins	+	-	+
Terpenoids	-	-	-

+ indicates presence while – indicates absence

**Figure 1:**
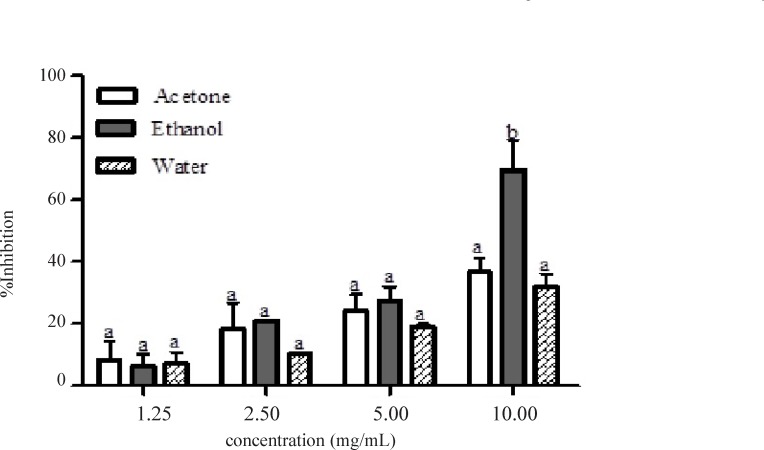
Percentage inhibition of α-amylase by different extracts of *Calotropis procera*

**Figure 2:**
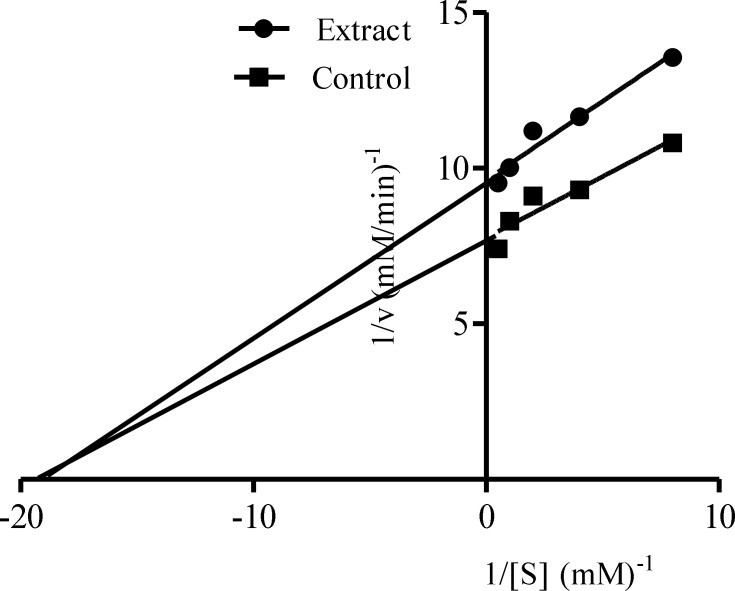
Mode of inhibition of α-amylase by ethanolic extract of *Calotropis procera*

Figure 1 showed the result of α-amylase inhibitory activities of the acetone, ethanolic and aqueous extracts of the *Calotropis procera*. At all concentrations tested, there were no significant differences (p > 0.05) among the values obtained for acetone, ethanolic and aqueous extracts of the plant except at 10.00 mg/mL where the percentage inhibition value for ethanolic extract was significantly different (p < 0.05) compared to the other extracts. Extrapolation of α-amylase inhibition effectiveness from the dose – response curve showed that ethanolic extract contained the most potent α-amylase inhibitor with an IC_50_ value of 7.80 mg/mL (Table 2). The mode of inhibition of α-amylase activity by the ethanolic extract of *Calotropis procera* leaf was determined using the Lineweaver-Burke plot which showed that the extract displayed a non-competitive inhibition of the enzyme activity (Figure 2).

**Table 2:** IC_50_ values of various extracts of *C. procera* against α-amylase and α-glucosidase.

**Extracts**	**IC**_50 _ **(mg/mL)**	
	**α-amylase**	**α-glucosidase**
Acetone	12.10 ± 1.22^a^	5.85 ± 0.36^a^
Ethanol	7.80 ± 0.53^b^	11.20 ± 0.90^b^
Water	15.75 ± 1.05^a^	3.25 ± 0.19^c^

Values carrying different superscripts along columns are significantly different (p < 0.05)

**Figure 3:**
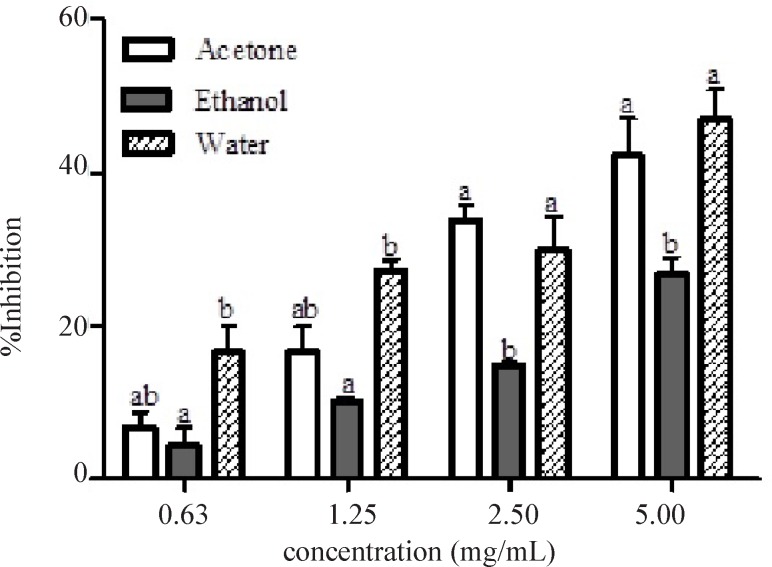
Percentage inhibition of α-glucosidase by different extracts of *Calotropis procera*

**Figure 4:**
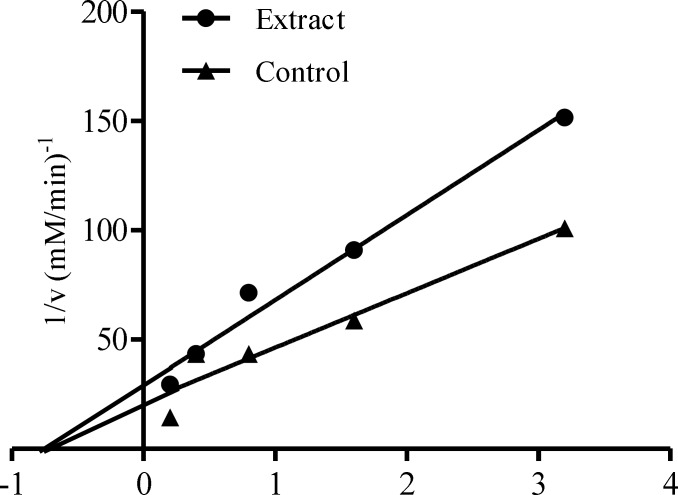
Mode of inhibition of α-glucosidase by aqueous extract of *Calotropis procera*

Figure 3 showed the α-glucosidase inhibitory activities of the leaf extracts of *Calotropis procera* at different concentrations (0.63 – 5.00.mg/mL). α-glucosidase inhibition by the different extracts was found to be dose dependent. At lower concentrations (0.63 and 1.25 mg/mL), there were significant differences (p < 0.05) between the ethanolic and aqueous extract in the inhibition of the enzyme but no significant difference (p > 0.05) between acetone and ethanolic nor between acetone and aqueous extracts. At higher concentrations, significant differences (p < 0.05) were noted between ethanolic extract when compared to either acetone or aqueous extract but there was no significant difference between acetone and aqueous extracts. Aqueous extract of this plant displayed the lowest IC_50_ (3.25 mg/mL) for the inhibition of α-glucosidase (table 2). The mode of inhibition of α-glucosidase by the aqueous extract of *Calotropis procera* obtained from Lineweaver-Burke plot was also non-competitive which is similar to that of α-amylase (Figure 4). 

## Discussion

Hyperglycemia is a state characterized by a rapid increase in blood glucose levels and is due to continuous hydrolysis of starch by pancreatic α-amylase and absorption of glucose by the intestinal α-glucosidases (31). One of the therapeutic approaches for decreasing postprandial hyperglycemia is to retard digestion of glucose by the inhibition of these carbohydrate hydrolyzing enzymes, α-amylase and α-glucosidase, in the digestive tract (31). Therefore, inhibition of these carbohydrate-hydrolyzing enzymes can significantly decrease postprandial hyperglycemia after a mixed carbohydrate diet and can be an important strategy in the management of diabetes mellitus (32).

In the present study, the results of the α-amylase and α-glucosidase inhibitory assay showed that the ethanolic and aqueous extracts of *Calotropis procera* are a mild inhibitor of α-amylase and a strong inhibitor of α-glucosidase, respectively. This is because higher concentration (7.80 mg/mL) of the extract is required to effectively inhibit α-amylase compared to the α-glucosidase which was obtained at 3.25 mg/mL. This is attested to by the respective IC_50_ generated for the inhibition of the enzymes by the extracts. This is in agreement with previous report that any medicinal plants that may be used as antidiabetic agents should be a mild inhibitor of α-amylase and strong inhibitor of α-glucosidase (33, 34). This has an edge over synthetic drugs such as acarbose which strongly inhibits both α-amylase and α-glucosidase, leading to side effects like abdominal distention, flatulence, meteorism and sometimes diarrhoea due to abnormal bacterial fermentation of undigested carbohydrates in the colon (33, 35).

The non-competitive inhibition displayed by both the ethanolic and aqueous extract of *Calotropis procera* towards both α-amylase and α-glucosidase, respectively, suggest that the active components of the extracts bind to a site other than the active site of the enzymes (36) and combine with both the free enzyme and the enzyme-substrate complex possibly interfering with the action of both. This affects the binding of the normal substrate to the enzyme due to conformational changes, thereby slowing down the breaking down of polysaccharides and disaccharides to glucose (37). Therefore, the concentration of glucose in the blood is maintained thereby controlling hyperglycemia and its complications. 

The inhibitory effect of the ethanolic and aqueous extracts of *Calotropis procera* on α-amylase and α-glucosidase, respectively, could be as a result of the phytochemicals present in them, which are flavonoids, steroids, saponins and tannins. Tannins have been reported to induce phosphorylation of the insulin receptors as well as translocation of glucose transporter 4 (GluT4), a major mediator of glucose removal from the circulation and a key regulator of whole-body glucose homeostasis. It also helps in the repression of the key gene responsible for adipogenesis thereby helping to reduce blood glucose level without increasing the adiposity (38). Flavonoids have also been reported to preserve β-cell integrity and function by scavenging free radicals in the system and therefore protect against the progression of diabetes mellitus (39). 

 It can be concluded from this study that out of the three extracts of *Calotropis procera* tested, the ethanolic extract displayed the most effective inhibition of α-amylase while aqueous extract displayed the most effective inhibition of α-glucosidase *in-vitro* and the mode of inhibition of both enzymes is the non-competitive one. The potent inhibitory activities of this plant may be due to the synergistic effect of phytochemicals present in it. 
